# Age-Dependent Changes in the Histogram of Apparent Diffusion Coefficients Values in Magnetic Resonance Imaging

**DOI:** 10.3389/fnagi.2013.00078

**Published:** 2013-11-19

**Authors:** Uwe Klose, Marion Batra, Thomas Nägele

**Affiliations:** ^1^MR Research Group, Department of Diagnostic and Interventional Neuroradiology, University Hospital Tübingen, Tübingen, Germany

**Keywords:** brain, atrophy, cerebrospinal fluid, magnetic resonance imaging, diffusion-weighted imaging

## Abstract

The aim of this study was to develop a fast method for estimating whether a brain volume loss is within the normal range for the respective age of the patient. A readout-segmented diffusion-weighted echo-planar imaging sequence was performed as part of the routine examination at a 3-T scanner. Data without (b0-image) and with diffusion weighting (1000 s/mm^2^) from 492 patients were examined (in the age from 3 to 89 years). One hundred and seventy-three data-sets had to be excluded due to brain lesions or to pathological enlarged cerebrospinal fluid spaces. In the remaining 319 data-sets, apparent diffusion coefficients (ADCs) values were calculated for all pixels exceeding a combined threshold in the diffusion-weighted data and in the non-diffusion-weighted data. The first part of the histogram represents pixels containing mostly brain tissue. The percentage of number of pixels in this part of the ADC histograms was evaluated for all patients and was correlated with the age of the patients. In all the areas examined, a monotone change of relative pixel numbers with the age of the patients was found. The reduction of the contribution of pixels containing mostly brain tissue accelerated with age and was found to be 0.18%/year in the age of 20, 0.34%/year in the age of 50, and 0.50%/year in the age of 80. The observed decrease of the relative number of pixels from the brain tissue with increasing age corresponds to previously published results based on more time-consuming 3-D measurements. The presented technique uses a conventional clinical sequence and might be helpful in deciding whether an observed brain volume loss in a patient is within the normal range for the age of the patient.

## Introduction

Several diseases of the brain lead to a volume reduction of brain tissue and an increase of cerebrospinal fluid (CSF) spaces (brain atrophy). However, brain volume reduction is also part of the normal aging process. The estimation whether an observed distribution of brain tissue and CSF is still within the normal range or is already pathological is a usual challenge for neuroradiologists. A possible way for such an estimation is through the acquisition of three-dimensional T1-weighted data-sets with high spatial resolution, which can be normalized to a standard brain and segmented into different brain compartments (Good et al., 2001; Taki et al., 2004; Grieve et al., 2005; Ziegler et al., 2012). In comparison with results from a large collection of normal subjects, a decision on normal or abnormal volume reduction of brain tissue for the respective age of the subject might be possible. However, this procedure is difficult to perform in clinical routine examinations. In this study, we examined whether a standard diffusion-weighted sequence can be used to get an estimation of normal or abnormal brain tissue volume.

Diffusion-weighted echo-planar imaging (DWI) sequences have been an important tool in clinical diagnostic Magnetic Resonance Imaging (MRI) of the brain for 20 years (Le Bihan et al., 1986; Moseley et al., 1990; Chien et al., 1992). On the other hand, the quality of DWI was, for a long time, limited by the necessity to acquire all raw data for whole images after single excitations. The matrix size of the widely used single-shot echo-planar imaging (EPI) sequence is often not more than 128 × 128 with a corresponding low spatial resolution.

The use of conventional multi-shot EPI sequences leads to severe image distortions induced by small physiological brain movements. These difficulties can now be avoided with the use of readout-segmented EPI (rs-EPI) sequences, which were introduced by Porter and Müller (2004) and are now successfully used at commercial MR scanners (Holdsworth et al., 2008, 2011; Porter and Heidemann, 2009; Heidemann et al., 2010; Naganawa et al., 2011; Morelli et al., 2013). We applied rs-EPI sequences in clinical routine examinations with an excellent image quality and evaluated whether apparent diffusion coefficient (ADC) histograms of data from DWI sequences can be used for an observation of age-dependent brain volume losses.

Diffusion-weighted echo-planar imaging sequences result in two images of excited slices: a diffusion-weighted image with a selected *b*-value (dw-image) and an image without diffusion weighting (*b*-value 0, therefore assigned as b0-image) with identical echo time. The image contrast of the b0-image is similar to usual T2-weighted images with a strong signal of the cerebrospinal fluid and a larger signal intensity of normal gray matter in comparison to normal white matter. In dw-images, CSF is dark and the signal of normal gray matter is again brighter than normal white matter. These properties are the reason for a strong difference between brain tissue and CSF in ADC maps, which can be used to examine general changes in the relationship between the volumes of brain tissue and CSF in the brain. In contrast to a recently published study of Watanabe et al. (2013) where ADC peak values and histogram widths were evaluated, we examined the relationship between the number of pixels with ADC values below and above a characteristic ADC value, which was determined in the average histogram of all patient data.

## Materials and Methods

All measurements were performed with a conventional 3 T MR whole-body scanner Skyra (Siemens Erlangen, Germany) equipped with a 20-channel head coil as part of the standard routine examination of patients with neuroradiological report requests. The patients were sent to the MR examination by several departments of the University Hospital of Tübingen including the departments of neurology, psychiatry, and neurosurgery. Informed consent was obtained after the nature of the procedure had been fully explained. The Ethics Committee of the University of Tübingen approved of this study.

Data were acquired between August 2012 and June 2013. In 492 patient examinations, the diffusion-weighted rs-EPI sequence was applied with identical measurement parameters: repetition time (TR) 6.3 s, echo time (TE) 73 ms, *b*-values 0 and 1000 mm/s^2^, matrix 224 × 224, FOV 230 mm, slice thickness 4 mm, slice distance 0.8 mm, 30 slices, number of segments of the segmented EPI sequence 5. All measurements were performed in axial orientation parallel to the line through anterior commissure and posterior commissure (AC-PC line). Data were checked visually and in the following cases data were excluded from the evaluation: data-sets with brain lesions due to infarctions or tumors (*n* = 82), with white matter lesions or large white matter defects (*n* = 32), with artifacts (e.g., due to a previous brain surgery, *n* = 8), with movement artifacts or wrong slice position (*n* = 4), and with incorrect selected compartments of the head coil (*n* = 1). Furthermore, the data-sets without diffusion weighting were scanned by an experienced neuroradiologist after sorting the data due to the patient age. In 46 cases, pathological enlargement of the CSF space was determined by the experienced neuroradiologist. These 46 data-sets were also excluded from the evaluation. The remaining number of patients was 319 (157 female, 162 male). Four age classes were built: 0–20 years (group 1, *n* = 30, 9 female, 21 male), 20–40 years (group 2, *n* = 72, 44 female, 28 male), 40–60 years (group 3, *n* = 115, 62 female, 53 male), and 60–90 years (group 4, *n* = 102, 42 female, 60 male). The age was calculated as difference between date of measurement and date of birth and used as a decimal number.

**Figure 1:**
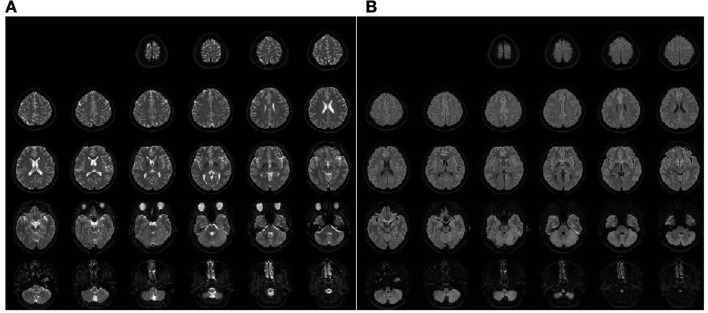
**Example of acquired data from one patient: b0-images (A) and dw-images (B)**.

Typical images with the applied sequence are shown in Figure 1. All 30 slices of the b0-images (Figure 1A) and the dw-images (Figure 1B) are shown.

The scaling of the acquired data was not consistent. Therefore, a histogram of values in the dw-images was evaluated for each patient on the basis of all 30 slices. The maximum of the smoothed histogram was estimated. The signal intensities of the acquired b0 and dw data were multiplied by a scale factor derived as the quotient of this median intensity and a chosen value of 250.

Noise pixels and pixels from the skull were excluded by applying a combined threshold derived from b0- and dw-images. For the remaining pixels, the ADC value was calculated and histograms of all ADC values were evaluated for each patient. The histograms were normalized to the number of pixel exceeding the noise threshold. This normalization compensates for different head sizes.

In the histograms, a characteristic ADC value was selected and the numbers of pixels below and those above this value were counted. The number of pixels below the characteristic ADC value were counted for different age and gender groups. Calculations were performed by computer program using MATLAB (MathWorks, Natick, MA, USA) written by one of the authors. The statistical significance of differences between the groups was calculated by the MATLAB function ttest2.

## Results

**Figure 2:**
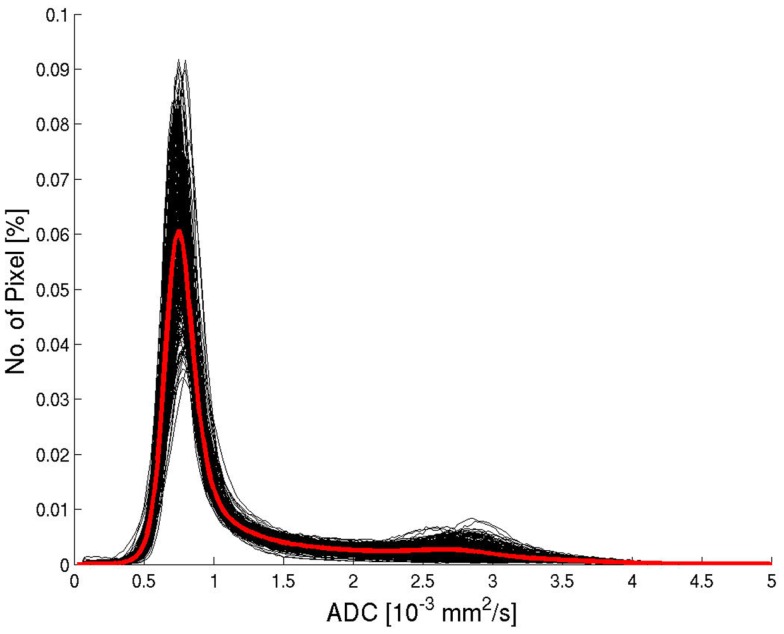
**Histograms of ADC values from all patients (black) and the average histogram (red)**.

**Figure 3:**
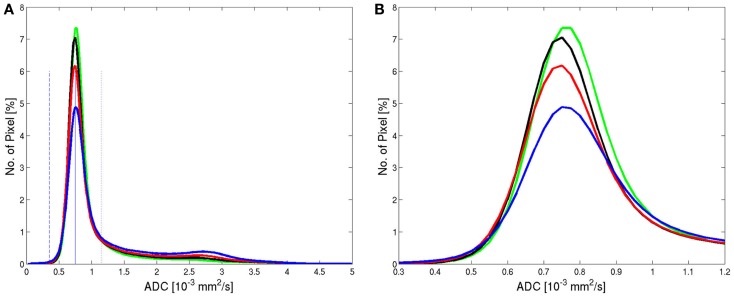
**Average histograms from all patients within the four age groups: <20 years (green, *n* = 30), 20–40 years (black, *n* = 72), 40–60 years (red, *n* = 115), >60 years (blue, *n* = 102) showed for the full range (A) and for a limited range (B) of ADC values**. The solid and dashed blue lines mark the center of the peak (solid) and the left basis (dashed) in the low ADC value range. The dotted blue line on the right side of the peak has the same distance of the peak as the dashed line and marks the upper value for integration.

All ADC histograms had a similar shape (Figure 2). The average histogram showed a clear peak for brain tissue for an ADC value of 0.75 × 10^−3^ mm^2^/s and a tail of larger values corresponding to pixels containing CSF or a considerable partial volume contribution from CSF. The average ADC histograms for the four selected age groups are shown in Figure 3. With increasing age, the relative number of pixels within the brain tissue peak decreases and the relative number of pixels within the CSF tail increases. A characteristic ADC value for the separation between pixels with brain tissue and with CSF was chosen as the mirrored basis of the brain tissue peak, which is clearly visible on the left side of the maximum (defined as the point, were a value of 1% of the maximum is reached). This characteristic ADC value was 1.15 × 10^−3^ mm^2^/s. After limitation of the range of ADC values in Figure 3B, changes of the ADC peak value can be seen: for young (≤20 years) and for old subjects (>60 years) the mean ADC peak value is larger than for subjects in the ages between 20 and 60 years.

**Figure 4:**
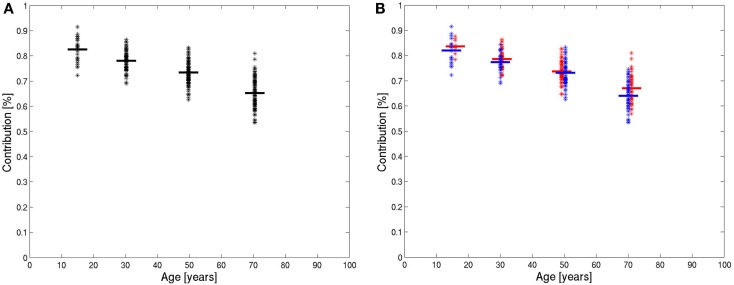
**Integrals of ADC histograms in the range of low ADC value below the characteristic value, shown in the four age groups of all patients (A) and in the four age groups of women (red) and men (blue) (B)**. The horizontal lines are the mean values.

The number of pixels in the histogram of each patient between ADC values of 0 and the characteristic value was evaluated in percent of the total number of pixels in the data-set above the used threshold. These numbers were averaged for all four age groups. The results are shown in Figure 4A. The differences between the groups were all significant with *p* < 0.001 after correction for multiple tests (*p*-values before correction: group 2 versus group 1: *p* = 7 × 10^−6^, group 3 versus group 2: *p* = 1 × 10^−11^, group 4 versus group 3: *p* = 5 × 10^−25^). In each age group, separate average values were calculated for female and male subjects (Figure 4B). In all age groups, the average of relative number of pixels in the brain compartment was slightly higher for females. In the first three age groups, this difference was not significant. In the group of subjects older than 60 years, a significant difference was found (*p* < 0.01).

**Figure 5:**
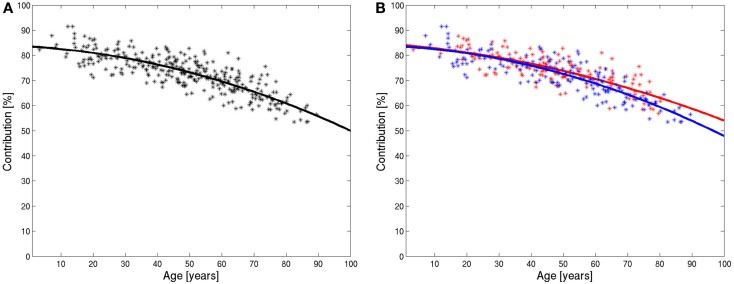
**Integrals of ADC histograms in the range of low ADC value below the characteristic value in dependence of the age of the patients and the fitted polynomial for all data (A) and for data from female (red) and male (blue) patients (B)**.

The value of the relative number of pixels in the brain compartment of each subject was marked as a point in Figure 5A against the age of the patient. The diagram shows decreasing values of the relative contribution of brain tissue pixels with age. The slope is not constant, therefore, the marked points were fitted by a polynomial of degree 2. The fitted polynomial is shown in Figure 5A. The equation of the polynomial is:

$$y=83.5+0.068x-0.0027x^{2}$$

The slope of this polynomial is −0.18%/year for the age of 20, −0.34%/year for the age of 50, and −0.50%/year for the age of 80.

The evaluation was repeated for female and male patients separately. The results are shown in Figure 5B. The decline of the contribution of brain tissue pixels is slightly stronger for men. The fitted polynomials are

$$\begin{array}{lll} y_{\mathrm{female}}=84.2-0.105x-0.0020x^{2} & \; & \\ y_{\mathrm{male}}=83.5-0.071x-0.0029x^{2} & \; & \end{array}$$

The decline is stronger for male patients and, the quadratic term, in particular, is higher for male patients, which indicate that the loss of relative brain tissue accelerates more with higher age. The values for the slope of the fitted polynomials at 20, 50, and 80 years are −0.18, −0.30, and −0.42% for females and −0.19, −0.36, and −0.53% for males.

## Discussion

MR or CT data-sets consist of large numbers of picture elements (pixel), which are characterized by a specific spatial position and signal intensity. In standard images, signal intensities are visualized as gray values at their respective spatial positions. Signal intensities of a whole data-set, of a single slice, or of a selected region of interest can also be analyzed as distribution of values. The properties of such a distribution can be described by a histogram. Such a histogram can be used to separate different structures. This procedure was very early examined for CT images, where standardized signal intensities exist (Hounsfield values). Hacker and Artmann (1978) used this technique to analyze the CSF content, Ratzka and Haubitz (1983) discriminates different brain tumors by histograms of CT data. The use of histograms in MR imaging is more difficult, because no absolute signal intensity values exist. Nevertheless, the shape of histograms can be used for tissue discrimination. An early example is the work of Pannizzo et al. (1992), who analyzed MR histograms to characterize multiple sclerosis lesions. This characterization of such lesions was improved by Mitchell et al. (1994), who performed a combined analysis of two data-sets with different weighting. They used two-dimensional histograms of proton density and T2-weighted images. Because both data-sets were acquired during the same measurement, no movement may disturb the spatial identity of pixels in both data-sets from the same position within the data matrix. The same condition is true for ADC and fractional anisotropy (FA) maps in diffusion tensor imaging (DTI) measurements. Lin et al. (2006) combined ADC and FA values in a two-dimensional histogram and differentiated patients with relapsing neuromyelitis optica from relapsing-remitting multiple sclerosis. In our study, we again used data with different weightings from a single measurement: the diffusion-weighted images and the non-diffusion-weighted image in a conventional DWI measurement. The histogram of data of ADC values from the whole brain showed a unique behavior. In a recently published study, Watanabe et al. (2013) found age-related changes of the ADC peak value in these histograms. Their results could be confirmed in this study (Figure 3B). In this study, the histogram was also used to discriminate signals from brain tissue and CSF. In order to compensate for the effect of different brain sizes, all evaluated pixel numbers were normalized to the sum of pixels without the pixels with background signal intensity. A similar approach was used by other researchers. La Joie et al. (2010) used the “total intracranial volume (TIV)” to normalize the volume of selected brain volumes. This TIV was calculated as the sum of the volumes of white matter, gray matter, and CSF. The reference value in this study needs no segmentation process and is directly evaluated from the ADC histogram. In this study, data from clinical routine measurements were used for an examination of the changes in the two-dimensional histogram with the age of the patients. Approximately one quarter of all patients had to be excluded because strong deformations due to different kinds of brain lesions, clear visible atrophic changes, or strong movement artifacts occurred. Although the remaining cohort of patients could not be assigned as normal control subjects, clear visible systematic changes of the ADC histograms with age were found. In contrast to usual studies with healthy controls, the examined cohort of subjects enclosed a large number of subjects older than 50 years.

An increase of the number of CSF pixels and a decrease of pixels from brain tissue with increasing age could be observed. This corresponds to previously published results. Previous estimations of changes of brain volume in relation to age are often based on T1-weighted 3-D measurement and a voxel-based morphometry analysis. In an early study, Good et al. (2001) found a decrease of relative gray matter volume of 0.17%/year for men and 0.15%/year for women (estimated from the Figures in Good et al., 2001) in combination with an slight increase of relative white matter. In the study of Taki et al. (2004), a decline of the relative gray matter of 0.24% for men and 0.2% for women is reported. Abe et al., found a decline of 0.2%/year in their study of 2006 (Abe et al., 2008) and 0.175%/year in 2010 (Abe et al., 2010). Grieve et al. (2005) found a weaker decline of gray matter (2.5 ml/year, TIV was 1516 ml, which means a slope of 0.16%/year) and a quadratic behavior of CSF volume: no change up to 40 years and a 0.2% increase between 60 and 80 years. In a recent study, Ziegler et al. (2012) found a reduction of relative gray matter volume by 0.23%/year, an increase of 0.05%/year for white matter, and a linear increase 0.17%/year for CSF [estimated from Figure S2 in Supplementary Material of Ziegler et al. (2012)]. The variance of these data shows that the segmentation performed by the voxel-based morphometry method does not lead to unique results. One reason for the uncertainty in the VBM method is the fact that most voxels contain a mixture of different tissues. The measured signal intensity is a sum of different compartments in this case, which might lead to errors in the assignment of voxels to specific tissues. Furthermore, the VBM technique needs to normalize the measured data to a standard brain. Therefore, all measured signal intensities have to be recalculated onto a new data grid by interpolation. This process again induces uncertainties near the borders of different structures.

In the present study, the original measured data were used for the evaluation of the ADC histogram without any interpolation. Only the scaling had to be adjusted as the signal intensities in MR imaging are only relative values. No transfer to the shape of the standard brain was performed. Instead, the different sizes of the examined heads were considered by the calculation of percentage of the selected pixels relative to the number of all evaluated pixels.

The relative number of pixels in the ADC histogram presents a strong dependence of the age of the examined persons. These numbers are not identical with the relative volume of brain structures because pixels with partial volume of brain tissue were not included. The applied evaluation technique does not allow a separation between white and gray matter as the peaks of these tissue types overlap.

Although there are strong differences in the presented approaches and the VBM technique used in previous studies on the age dependence of MR data, some of the results are comparable.

The results from Taki et al. (2004) and those from other groups for the decrease of gray matter with age are near the slope of the fitted polynomials found for subjects in the age between 20 and 30 years in this study.

Good et al. (2001) reported a decline of 0.17 and 0.15%/year for relative gray matter in men and in women. This is slightly less than the slopes which were found in this study for subjects 20 years of age (0.19 and 0.18%). The slopes of the decline of relative brain tissue in this study are also steeper than in the recent study from Ziegler et al. (2012). The reason might be that the exclusion criteria in the study of Ziegler et al., and other studies were much stronger. Here we excluded only subjects with clearly visible pathological changes (beside the exclusion due to artifacts or wrong slice positioning). Nevertheless, the obtained results show a clear decline of the relative numbers of brain pixels with a limited variation, and thus, this observation may be used to differentiate a pathological atrophical reduction of brain tissue from the normal aging effect. The comparison of results from single patients with the presented group analysis might lead to improvements of the neuroradiological diagnostics of patients with neurodegenerative and demential diseases.

A selective evaluation of different brain regions in patients with specific patterns of atrophy, such as Alzheimer’s disease, frontotemporal dementia, or corticobasal degeneration, is planned to be introduced in this technique by pre-selection of spatial brain regions.

In conclusion, the presented technique provides a simple possibility to show the age dependence of MR signal intensities within the brain. The presented technique is applicable in the clinical environment. It uses data from a sequence which is often part of the standard diagnostic procedure, and no data segmentation or other data transformation apart from rescaling is necessary.

## Author Contributions

Uwe Klose developed the method of data analysis and wrote the main part of the manuscript. Marion Batra was the supervisor of the patient examinations and decided to exclude patient data from the evaluation due to clinical findings. Thomas Nägele defined the protocol of the patient examinations and excluded patients with pathologically enlarged CSF spaces.

## Conflict of Interest Statement

The authors declare that the research was conducted in the absence of any commercial or financial relationships that could be construed as a potential conflict of interest.
